# Targeting the isoprenoid pathway in choleste biosynthesis: An approach to identify isoprenoid biosynthesis inhibitors

**DOI:** 10.1002/ardp.202400807

**Published:** 2025-02-13

**Authors:** Maximilian Liebl, Florian Olander, Christoph Müller

**Affiliations:** ^1^ Department of Pharmacy—Center for Drug Research Ludwig‐Maximilians‐Universität München Munich Germany

**Keywords:** 6‐fluoromevalonate, GC‐MS, isoprenoid trafficking, pre‐squalene pathway, pyrophosphate

## Abstract

The development of novel cholesterol biosynthesis inhibitors is a task of major concern due to the diverse roles of cholesterol and its precursors in physiological processes. Therefore, appropriate screening assays are required, which can be used to identify and quantify specific inhibitors targeting the desired enzyme. Here, we developed a whole‐cell screening assay based on a HL60 cell line, which can be used to characterize inhibitors interacting with enzymes of the isoprenoid part of cholesterol biosynthesis. Due to the change of the isoprenoid pattern under enzyme inhibition, an identification of the targeted enzyme is possible. With the described assay, we can distinguish between free and pyrophosphorylated isoprenoids after enzymatic cleavage in cellular and extracellular matrices. The approach was validated in line with the European Medicines Agency guideline on bioanalytical method validation. As proof of concept, literature‐described inhibitors of the isoprenoid pathway were tested. We characterized the effect of 11 isoprenoid biosynthesis inhibitors, and we identified 6‐fluoromevalonate as an isopentenyl pyrophosphate isomerase inhibitor, a biological activity that was previously unknown. Furthermore, isoprenoid patterns revealed that, independent of the analyzed matrix, the predominant form of the detected isoprenoids were dephosphorylated isoprenoids and only small amounts were present as pyrophosphates.

## Graphical abstract

A fully validated assay is reported that can be used to identify inhibitors of isoprenoid biosynthesis by gas chromatography‐mass spectrometry (GC‐MS). The free isoprenoids can be distinguished from their pyrophosphorylated species. From 11 literature‐described isoprenoid biosynthesis inhibitors, eight influenced the isoprenoid patterns of HL60 cells. The assay can be applied to cellular and extracellular matrices, making it an ideal approach for isoprenoid trafficking.

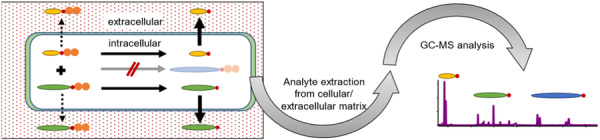

## 1 INTRODUCTION

Sterols are ubiquitous in eukaryotic cells and play an important role for membrane stability, flexibility, and rigidity.^[^ ^1^ ^]^ They are precursors of bile acids, as well as secosteroids like Vitamin D3 and have an essential role in the endocrine system.^[^ ^2^ ^]^ Their biosynthetic precursors, the isoprenoids, are not less important as they support several physiological processes. Next to the formation of dolichols, ubiquinone, heme A, and fungal carotenoids, one of their key roles is their participation in multiple posttranslational modification reactions of small GTPases such as Ras, Rab, Rho, and Rac.^[^ ^3^, ^4^, ^5^, ^6^ ^]^ Isoprenoids can even modify specific transfer RNA (*t*RNA) sections.^[^ ^7^ ^]^

**Figure 1:**
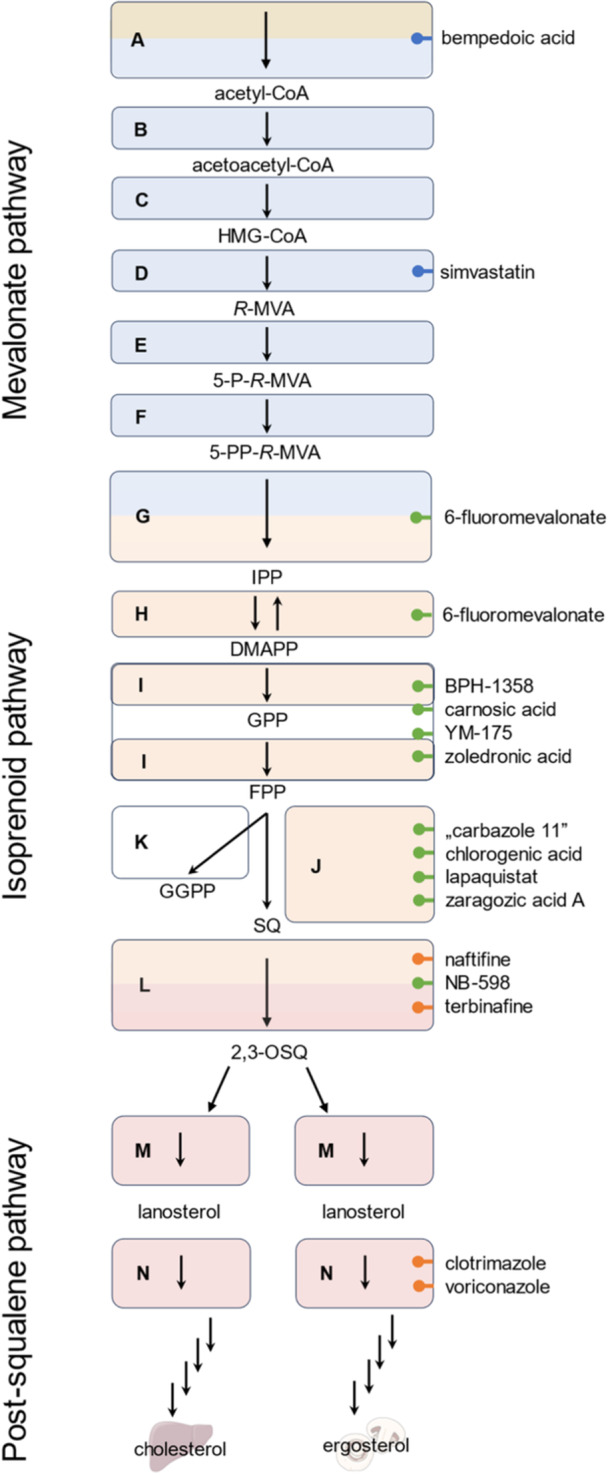
The subpathways of sterol biosynthesis with a spotlight on the presqualene part. Drugs for treating hyperlipidemia are depicted in blue bullets, drugs for antifungal therapy (orange bullets), and postulated experimental isoprenoid biosynthesis inhibitors (green bullets). Blue boxes contain enzymes of the mevalonate pathway, orange boxes contain enzymes of the isoprenoid pathway, white boxes contain enzymes that are not involved in sterol biosynthesis, red boxes contain enzymes of the post‐squalene pathway. Two‐colored boxes contain enzymes that interconnect two subpathways. Intermediates: acetyl coenzyme A (acetyl‐CoA); acetoacetyl coenzyme A (acetoacetyl‐CoA); 3‐hydroxy‐3‐methylglutaryl coenzyme A (HMG‐CoA); *R*‐mevalonate (*R*‐MVA); mevalonate‐5‐*R*‐phosphate (5‐P‐*R*‐MVA); mevalonate‐5‐*R*‐pyrophosphate (5‐PP‐*R*‐MVA); isopentenyl pyrophosphate (IPP); dimethylallyl pyrophosphate (DMAPP); geranyl pyrophosphate (GPP); farnesyl pyrophosphate (FPP); geranylgeranyl pyrophosphate (GGPP); squalene (SQ); 2,3‐oxidosqualene (2,3‐OSQ). Enzymes: Adenosine triphosphate citrate synthase (ATP citrate synthase, (**A**); acetoacetyl‐CoA thiolase (**B**); HMG‐CoA synthase (**C**); HMG‐CoA reductase (**D**); mevalonate‐5‐kinase (**E**); phosphomevalonate kinase (**F**); mevalonate pyrophosphate decarboxylase (**G**); IPP isomerase (**H**); FPP synthase (**I**); squalene synthase (**J**); GGPP synthase (**K**); squalene epoxidase (**L**); lanosterol synthase (**M**); lanosterol 14α‐demethylase (**N**). Parts of this figure were created using Servier Medical Art templates, licensed unde a Creative Commons Attribution 3.0 Unported License (https://smart.servier.com, accessed October 2024).

**Figure 2:**
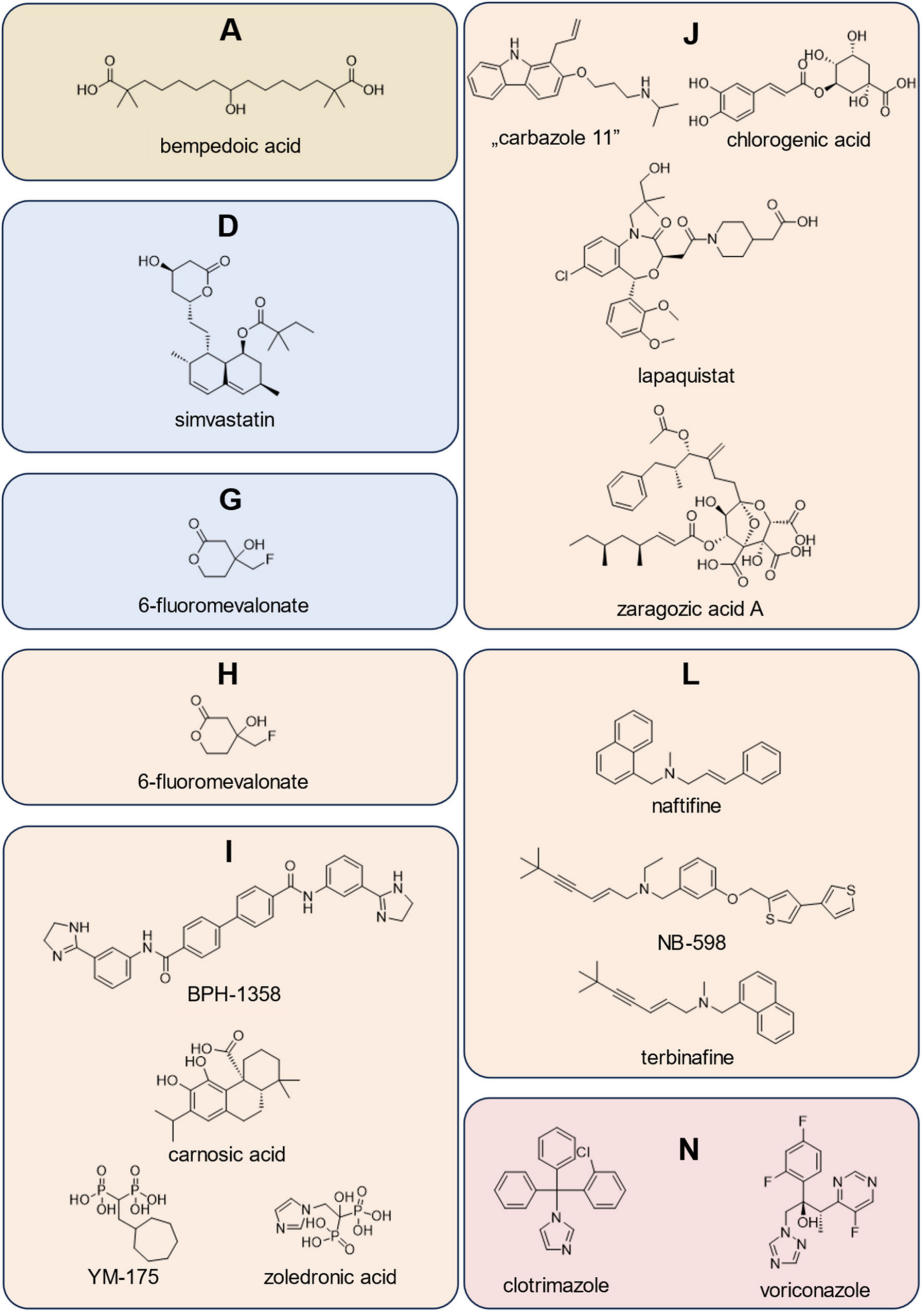
Sterol biosynthesis inhibitors with a focus on the isoprenoid pathway. Colors show the subpathway where the putative inhibited enzyme belongs to: brown (before mevalonate pathway); blue (mevalonate pathway); orange (isoprenoid pathway); red (post‐squalene pathway). Putative target enzymes: Adenosine triphosphate citrate synthase (ATP citrate synthase, **A**); HMG‐CoA reductase (**D**); mevalonate pyrophosphate decarboxylase (**G**); isopentenyl pyrophosphate isomerase (**H**); farnesyl pyrophosphate synthase (**I**); squalene synthase (**J**); squalene epoxidase (**L**); lanosterol 14α‐demethylase (**N**). HMG‐CoA, 3‐hydroxy‐3‐methylglutaryl coenzyme A.

In general, sterol biosynthesis can be divided into two major parts, the pre‐ and the post‐squalene pathway. While in mammalian cells cholesterol is the main product of its sterol biosynthesis, the fungal equivalent is ergosterol (Figure 1). Plants produce several main sterols including sitosterol, stigmasterol, and campesterol.^[^ ^8^ ^]^ The formation of the post‐squalene pathway intermediates is catalyzed by organism‐specific enzymes that often distinguish in their affinity for drug substances, thereby making them a selective target for treatment (Figure 1). One class of inhibitors targeting the enzymes of the post‐squalene pathway are allylamines such as terbinafine and naftifine (Figure 2, **L**) which demonstrate a high affinity toward fungal squalene epoxidase (SE, Figure 1, **L**). In contrast, the mammalian SE (EC 1.14.14.17) is not affected by these compounds at concentrations that are therapeutically relevant.^[^ ^9^ ^]^ Conversely, the experimental inhibitor NB‐598 (Figure 2, **L**) is a selective and potent inhibitor of the mammalian SE, only displaying minimal effects on the corresponding yeast enzyme.^[^ ^9^ ^]^ A comparable degree of selectivity is observed with the azole antimycotics clotrimazole^[^ ^10^ ^]^ and voriconazole^[^ ^11^ ^]^ (Figure 2, **N**) that only affect mammalian lanosterol 14α‐demethylase (**N**, EC 1.14.14.154) at concentrations exceeding their therapeutic application limit.

The up to now only poorly investigated presqualene pathway of sterol biosynthesis can be further divided into a mevalonate and an isoprenoid section that results in squalene, the branching point of sterol biosynthesis in diverse organisms (fungi, plants, mammals) (Figure 1). Statins, for example, simvastatin (Figure 2, **D**), inhibitors of the enzyme 3‐hydroxy‐3‐methyl‐glutaryl‐coenzyme A (HMG‐CoA) reductase (Figure 1 **C**, EC 2.3.3.10), are the most prominent drugs affecting the presqualene pathway. They are used as first‐line therapy in hyperlipidemia. Besides statins no further drugs target enzymes of the mevalonate section of cholesterol biosynthesis. The most common disadvantage of statin treatment is the occurrence of adverse effects, which prevent up to 29% of the patients from using statins at the doses required for therapeutic efficacy.^[^ ^12^, ^13^, ^14^ ^]^ Side effects, like the primary muscular complaints are attributable to the impact of statins on skeletal muscle, wherein downstream products of HMG‐CoA are essential for the normal functioning of muscle cells.^[^ ^12^ ^]^ Therefore, in 2020 bempedoic acid, (Figure 2, **A**) was approved by the Food and Drug Administration (FDA) as well as by the European Medicines Agency (EMA) for the treatment of hypercholesteremia in statin‐intolerant patients.^[^ ^14^ ^]^ This prodrug is a first‐in‐class drug that is selectively activated in liver tissue, where it affects cholesterol biosynthesis by inhibiting ATP citrate lyase (Figure 1 **A**, EC 2.3.3.8), an enzyme providing acetyl‐CoA, the precursor of the mevalonate pathway.^[^ ^14^, ^15^ ^]^ A less prominent substance targeting the isoprenoid pathway is 6‐fluoromevalonate (Figure 2, **G**, **H**) which has originally been described as an inhibitor of mevalonate pyrophosphate decarboxylase (Figure 1 **G**, EC 4.1.1.33), the enzyme converting mevalonate‐5‐*R*‐pyrophosphate (5‐PP‐*R*‐MVA) into isopentenyl pyrophosphate (IPP).^[^ ^16^ ^]^ In 2011, Henneman et al.^[^ ^17^ ^]^ identified further accumulating intermediates in their assays after 6‐fluoromevalonate treatment. Next to 5‐PP‐*R*‐MVA, the upstream precursors mevalonate‐5‐*R*‐phosphate (5‐P‐*R*‐MVA) and *R*‐mevalonate (*R*‐MVA), as well as the downstream isoprenoid pyrophosphates, IPP, and/or dimethylallyl pyrophosphate (DMAPP) were identified. The authors were unable to differentiate between IPP and DMAPP due to their analytical setup.

The group of nitrogen‐containing bisphosphonates (NBP), for example, zoledronic acid and YM‐175 (Figure 2, **I**)^[^ ^18^ ^]^ affect the isoprenoid pathway by inhibiting farnesyl pyrophosphate synthase (FPPS; Figure 1 **I**, EC 2.5.1.1/EC 2.5.1.10), resulting in a lack of farnesyl pyrophosphate (FPP) and geranylgeranyl pyrophosphate (GGPP). Those two substrates are essential in posttranslational modification reactions of GTPases, small signaling proteins, regulating various cellular processes that are important for osteoclast function.^[^ ^4^ ^]^ In addition, FPP and GGPP were proven to exhibit a pivotal role in cancer cell signaling and expansion.^[^ ^19^, ^20^ ^]^ Due to the inhibitory effect on FPPS, NBPs affect sterol biosynthesis and showed to have beneficial effects on total sterol levels in cases of hyperlipidemia.^[^ ^21^ ^]^ The reason why NBPs cannot be used for the treatment of hyperlipidemia is their poor membrane permeability in combination with a high affinity toward bone minerals, which explains the interest in non‐NBP inhibitors of FPPS.^[^ ^4^, ^22^ ^]^ One attempt to identify new classes of inhibitors was therefore made by Lindert et al.^[^ ^22^ ^]^ using in silico screening, where several bisamidines, including BPH‐1358 (Figure 2, **I**) as the most promising one, showed their potential as FPPS inhibitors. Carnosic acid, also a non‐NBP compound (Figure 2, **I**), was identified by Han et al.,^[^ ^23^ ^]^ who investigated the molecular targets of rosemary and sage extract that are known for their antitumor, antimicrobial, and cholesterol‐lowering effects.

The enzyme squalene synthase (SQS; Figure 1 **J**, EC 2.5.1.21) is a further promising target in the pre‐squalene pathway. With chlorogenic acid (Figure 2, orange) a naturally occurring SQS inhibitor was extracted from the fruit of *Prunus mume* by Choi et al.^[^ ^24^ ^]^ The authors determined an inhibitory effect of the substance on pig liver SQS in nanomolar concentrations. An additional hit was ”carbazole 11” (Figure 2, **J**), which was the most potent compound out of several propylamine derivatives, to inhibit SQS in HepG2 cells resulting in lower total plasma cholesterol levels in rats after oral dosing.^[^ ^25^ ^]^ However, the best described inhibitors of SQS are the three closely related zaragozic acids (ZA) A/B/C which were isolated from the fungal species *Sporominella intermedia* and *Leptodontium elatius* in the 1990s. The most potent of them, ZA A, was therefore named squalestatin.^[^ ^26^ ^]^ Based on their structure, new SQS inhibitors were designed to treat hyperlipidemia and lapaquistat (TAK‐475; Figure 2, **J**) was the most promising drug candidate that could have been the first SQS inhibitor on the market. However, late clinical studies and the further development were terminated in 2008 when a dose‐dependent increased liver toxicity was observed for lapaquistat.^[^ ^27^, ^28^ ^]^ One explanation for the observed hepatotoxicity could be an increase of the metabolic precursor farnesol, which can affect the cell cycle and induce apoptosis.^[^ ^20^, ^29^ ^]^ The group of Nagashima et al.^[^ ^29^ ^]^ therefore developed an SQS knockout mouse model in which they observed transient liver dysfunction which they correlated with increased farnesol concentrations. Nevertheless, the role of SQS inhibitors as sterol biosynthesis inhibitors as well as therapeutic targets for previously seemingly unrelated diseases (like Alzheimer's disease) is of major concern.^[^ ^30^ ^]^

One of the biggest challenges in developing potential drug candidates targeting sterol biosynthesis is to guarantee the selectivity of a new compound to a desired target. Therefore, cell‐based in vitro assays are necessary where in a quick and easy setup, multiple compounds can be tested for their activity on numerous enzymes. For the screening of distal sterol biosynthesis, several potent approaches exist.^[^ ^31^, ^32^, ^33^, ^34^, ^35^ ^]^ Nevertheless, the pre‐squalene pathway was disregarded in these approaches so far. For this reason, we recently developed an assay that gives us an enlarged view on ergosterol biosynthesis capturing the isoprenoid pathway.^[^ ^36^ ^]^ In this work, we extended our approach to mammalian isoprenoid biosynthesis by validating the assay on human leukemia cells (HL60) using the EMA guideline on bioanalytical method validation.^[^ ^37^ ^]^ In combination with our HL60‐based assay for the identification of distal cholesterol biosynthesis inhibitors,^[^ ^31^ ^]^ we are expanding our analytical scope for the analysis of inhibitors in the pre‐squalene pathway. We can evaluate “hits” on mammalian or fungal sterol biosynthesis and thereby provide information about organism specificity. In addition, our assay can be used to analyze isoprenoid and isoprenoid pyrophosphate trafficking between cellular and extracellular matrices. As proof of concept, we characterized, to the best of our knowledge, the most important predictive drugs, and other well‐known experimental inhibitors of the isoprenoid pathway of cholesterol biosynthesis on HL60 cells. We determined substance specific isoprenoid patterns by analyzing intracellular and extracellular isoprenoid and isoprenoid pyrophosphate levels. Furthermore, we determined IC_50_ values, based on the total reduction of cholesterol biosynthesis, to characterize the cholesterol‐lowering activity of the compounds of interest.

## 2 RESULTS AND DISCUSSION

### 2.1 Validation results cellular matrix

#### 2.1.1 Linearity and quantification limits

For all analytes, a linear fit (R^2^ ≥0.993) was determined for the matrix‐matched calibration (Table 1, linearity). The linear range was from 2.5 to 1000 ng/mL. Therefore, the lower limit of quantification (LLOQ) was set at 2.5 ng/mL for all isoprenoids. Squalene was quantified reliably in concentrations from 10 to 1000 ng/mL.

**Table 1:** Validation data for cellular matrix.

Validation parameter	Value	Concentration	Analytes						
			Isoprenol	Prenol	Geraniol	Squalene	Farnesol	Geranyl‐geraniol	Average
Linearity	Linear range (ng/mL)	–	2.5–1000	2.5–1000	2.5–1000	10–1000	2.5–1000	2.5–1000	–
	R^2^	–	≥0.993	≥0.996	≥0.996	≥0.995	≥0.998	≥0.997	–
Matrix effects	Matrix factor	Low*	0.75	1.13	1.03	3.99	1.08	1.56	1.59
		High	0.92	0.96	1.01	1.06	1.09	1.07	1.02
	RSD (%)	Low*	4	1	5	3	4	7	4
		High	3	3	3	6	5	5	4
Method accuracy	–	LLOQ*	1.00	0.82	1.19	1.20	0.92	0.86	1.00
		Low*	0.98	0.90	1.01	1.15	0.91	0.97	0.99
		Medium	1.00	1.05	1.05	1.01	1.00	0.98	1.02
		High	0.98	1.00	1.01	0.98	0.99	0.98	0.99
		Average	0.99	0.95	1.07	1.09	0.95	0.95	–
Method precision	(%)	LLOQ*	4	3	5	3	10	10	6
		Low*	6	3	4	3	15	13	7
		Medium	1	2	4	2	4	4	3
		High	3	4	3	5	5	4	4
		Average	4	3	4	3	9	8	–
Recovery	Recovery (SD) (%)	LLOQ	–	91 (5)	–	–	150 (17)	50 (8)	97 (10)
		Low	–	77 (3)	–	–	133 (6)	35 (8)	82 (6)
		Medium	–	76 (4)	–	–	160 (7)	28 (2)	88 (4)
		High	–	73 (2)	–	–	166 (2)	34 (2)	91 (2)
		Average	–	79 (3)	–	–	152 (8)	37 (5)	–

*Note*: LLOQ, 2.5 ng/mL; low, 7.5 ng/mL; medium, 500 ng/mL; high, 1000 ng/mL. LLOQ* (squalene), 10 ng/mL; low* (squalene), 25 ng/mL.

Abbreviations: ‐, not determined/applicable; RSD, relative standard deviation; SD, standard deviation; R^2^, linear fit.

#### 2.1.2 Matrix effects

Due to the absence of squalene free matrix, pooled sample matrix was used to generate a constant level of squalene in all samples. Especially in the low quality control (QC; 7.5/25 ng/mL) samples (Table 1, matrix effects), matrix induced signal changes were detected. The highest influence of cellular matrix on signal areas was determined for squalene where a high MF of 3.99 was detected, but the signal of geranylgeraniol was also increased in the presence of cellular matrix (matrix factor [MF] = 1.56). The opposite effect was detected for isoprenol in the low QC samples, where the signals were decreased (MF = 0.75). In the high QC (1000 ng/mL) samples, only the signals of isoprenol were decreased (MF = 0.92), whereas the remaining analytes were not influenced significantly (average MF = 1.02). In accordance with the EMA guideline, RSD was ≤7% in both concentrations and therefore fits the guideline's limits of <15%.

#### 2.1.3 Method accuracy and precision

Method accuracy was evaluated at four concentrations: LLOQ (2.5/10 ng/mL), low (7.5/25 ng/mL), medium (500 ng/mL), and high (1000 ng/mL). For each concentration, six QC samples were analyzed. The accuracy was determined by comparing the back‐calculated concentration to the theoretical concentration of its respective level. The average accuracy was between 0.99 and 1.02 in the respective levels (Table 1, method accuracy). Considering the different analytes, average values reach from 0.95 (prenol, farnesol, geranylgeraniol) to 1.09 (squalene). In addition, none of the single values exceeded the recommended limits of 1.00 ± 0.20 (LLOQ) and 1.00 ± 0.15 (remaining levels). Only squalene reached 1.20 at LLOQ and 1.15 at the low concentration. Method precision was evaluated within the same batch of samples and should be within 20% (LLOQ) or 15% (remaining levels). Method precision was ≤9% (farnesol) considering the average precision of the different analytes. Single QC levels did not exceed an RSD of 15% (Table 1, method precision).

#### 2.1.4 Recovery of isoprenoids from isoprenoid pyrophosphates

Recovery was assessed as an additional validation parameter, due to the use of different matrices that could probably influence extraction efficiency. Solutions of three pyrophosphates (DMAPP, FPP, and GGPP) were spiked in equimolar concentrations to LLOQ, low QC, medium QC, and high QC samples and compared with respective QC standards. The mean recovery ranged from 82% to 97% for the different concentrations (Table 1, recovery). While the average recovery for prenol, the alcohol originating from DMAPP, was 79%, the mean recovery for farnesol (originating from FPP) was 152%. For geranylgeraniol (originating from GGPP) the recovery was 37%. Even though recovery values differed from the expected 100%, they were comparable and constant to our previous results for *Saccharomyces cerevisiae* and *Aspergillus fumigatus* cell matrices (prenol 107% and 85%, farnesol 133% and 109%, geranylgeraniol 44% and 37%).^[^ ^36^ ^]^ In addition, recovery values were precise with SDs ≤8%, except farnesol at the LLOQ (17%).

### 2.2 Validation results extracellular matrix

#### 2.2.1 Linearity and quantification limits

The matrix‐matched calibration of lyophilized extracellular matrix was linear in a range from 2.5 to 1000 ng/mL for all analytes including squalene (Table 2, linearity). Linear correlation was given with R^2^ ≥0.994. The LLOQs were determined at 2.5 ng/mL.

**Table 2:** Validation data for extracellular matrix.

			Analytes						
Validation parameter	Value	Concen‐tration	Isoprenol	Prenol	Geraniol	Squalene	Farnesol	Geranyl‐geraniol	Average
Linearity	Linear range (ng/mL)	–	2.5–1000	2.5–1000	2.5–1000	2.5–1000	2.5–1000	2.5–1000	–
	R^2^	–	≥0.997	≥0.996	≥0.999	≥0.998	≥0.994	≥0.990	–
Matrix effects	Matrix factor	Low	1.22	1.03	1.26	1.63	1.20	1.37	1.29
		High	1.00	1.01	1.05	0.96	1.08	1.11	1.03
	RSD (%)	Low	7	7	9	10	9	9	9
		High	9	8	9	9	9	8	9
Method accuracy	–	LLOQ	1.13	1.11	1.29	0.86	1.04	1.05	1.08
		Low	1.01	1.09	1.09	0.98	0.88	0.94	1.00
		Medium	1.05	1.09	1.15	1.11	1.05	1.05	1.08
		High	1.00	1.04	1.09	1.11	1.07	1.10	1.07
		Average	1.05	1.08	1.16	1.01	1.01	1.04	–
Method precision	–	LLOQ	4	4	3	4	8	5	5
		Low	4	6	2	6	3	7	5
		Medium	1	1	1	0	1	1	1
		High	1	1	2	1	1	1	1
		Average	3	3	2	3	3	4	–
Recovery	Recovery (SD) (%)	LLOQ	–	86 (21)	–	–	140 (12)	51 (4)	93 (13)
		Low	–	82 (5)	–	–	136 (8)	41 (8)	86 (7)
		Medium	–	78 (2)	–	–	137 (5)	26 (1)	81 (3)
		High	–	72 (2)	–	–	141 (4)	27 (1)	80 (2)
		Average	–	80 (7)	–	–	139 (7)	37 (4)	–

*Note*: LLOQ, 2.5 ng/mL; low, 7.5 ng/mL; medium, 500 ng/mL; high, 1000 ng/mL.

Abbreviations: ‐, not determined/applicable; RSD, relative standard deviation; SD, standard deviation; R^2^, linear fit.

#### 2.2.2 Matrix effects

The influences of the extracellular matrix on signal intensities were also investigated using a pooled matrix. For low QC (7.5 ng/mL) samples, effects could be detected in all samples (average MF = 1.29; Table 2, matrix effects). The least affected analyte was prenol (MF = 1.03), whereas squalene showed the highest differences compared with the *n‐*hexane standards (MF = 1.63). In the high QC (1000 ng/mL) samples, the influence of the matrix on signal areas was low with an average MF of 1.03. The highest influence of matrix was determined for geranylgeraniol (MF=1.11). Although, concentration‐dependent matrix effects were present, the observed RSD in both levels was ≤10% and below the required limit of 15%.

#### 2.2.3 Accuracy and precision

Accuracy was tested using six QC samples of four different concentrations LLOQ (2.5 ng/mL), low (7.5 ng/mL), medium (500 ng/mL), and high (1000 ng/mL). The average accuracy was between 1.00 and 1.08 in the separate levels (Table 2, method accuracy). Considering the different analytes, average values reached from 1.01 (squalene, farnesol) to 1.16 (geraniol). In the LLOQ QC samples, the recommended range (1.00 ± 0.20) was exceeded for geraniol (1.29). The analysis of all further QC samples gave accuracy values between 0.86 and 1.15 and therefore met the requirements. Method precision was ≤4% considering the average precision of the different analytes. All QC samples did not exceed an SD of 8% (Table 2, method precision).

#### 2.2.4 Recovery of isoprenoids from isoprenoid pyrophosphates

Recovery of the method was tested in an equivalent procedure to the cellular matrix. The mean recovery was between 80% and 93% (Table 2, recovery). Considering the individual analytes, the mean recovery for prenol from DMAPP was 80%. Farnesol was recovered by 139% from FPP in average and geranylgeraniol from GGPP by 37%. Overall samples, precision is given with SD values ≤12%, except for prenol at the LLOQ (21%). The results are consistent with the results obtained from the cellular matrix (see Section 2.1.4).

### 2.3 Biological results

#### 2.3.1 Characterization of inhibitors

Inhibitory effects on the isoprenoid pathway were identified for eight of the 16 tested substances (Table 3, qualitative screening). In addition, effects on the post‐squalene pathway were identified for two compounds according to Müller et al.^[^ ^31^ ^]^ 6‐Fluoromevalonate led to an accumulation of isoprenol in our screening. Given the absence of prenol, the second possible C_5_ isoprenoid described by Henneman et al.,^[^ ^17^ ^]^ we were able to confirm a selective inhibition of IPP isomerase (**H**) by this compound. The group of Muehlbacher et al.^[^ ^38^ ^]^ described a fluorinated IPP (3‐(fluoromethyl)but‐3‐en‐1‐pyrophosphate) to be a selective inhibitor of IPP isomerase. However, 6‐fluoromevalonate was not identified to be a prodrug for 3‐(fluoromethyl)but‐3‐en‐1‐pyrophosphate (data not shown). Both NBPs, zoledronic acid, and YM‐175, led to an accumulation of isoprenol, prenol, and geraniol by inhibiting FPPS (**I**). The inhibitors ZA and lapaquistat led to an accumulation of farnesol and could be confirmed as inhibitors of SQS (**J**). For squalene epoxidase (**L**) we were able to confirm the three inhibitors NB‐598, terbinafine, and naftifine, that led to an accumulation of squalene. The inhibitors of the mevalonate pathway, bempedoic acid, and simvastatin that target ATP citrate synthase (**A**) or HMG‐CoA reductase (**D**) respectively, as well as the post‐squalene pathway inhibitors clotrimazole and voriconazole, inhibiting lanosterol 14α‐demethylase (**N**) showed no changes on the isoprenoid pattern. Furthermore, the postulated effect of BPH‐1358 and carnosic acid on FPPS (**I**, accumulation of DMAPP) as well as the effects of “carbazole 11” and chlorogenic acid on SQS (**J**, accumulation of FPP) could not be confirmed. In addition to the qualitative screening, IC_50_ values were determined (Table 3, quantitative screening). Only BPH‐1358 could not be fully characterized, which was due to the low solubility of the substance above concentrations of 50 µM. Bempedoic acid and 6‐fluoromevalonate did not show any impact on total cholesterol levels, irrespective of the incubated concentration (Table 3, quantitative screening). Consequently, a specific IC_50_ value could not be determined. For all three inhibitors of squalene epoxidase (**L**), IC_50_ values were determined. The most potent one was NB‐598, a described inhibitor of mammalian squalene epoxidase, with an IC_50_ value of 0.006 µM, whereas the remaining two inhibitors, terbinafine and naftifine, inhibitors of fungal squalene epoxidase, were only effective in higher concentrations with IC_50_ values between 0.540 and 1.9 µM. Also, two inhibitors of SQS (**J**), lapaquistat and ZA, were effective inhibitors of the cholesterol biosynthesis with IC_50_ values ranging from 2 to 5 µM. The highest IC_50_ values were determined for the two NBPs, YM‐175 (337 µM) and zoledronic acid (225 µM). Even though no effect on the isoprenoid pathway or post‐squalene pathway enzymes was detected, “carbazole 11” was identified as an effective inhibitor of total cholesterol biosynthesis with an IC_50_ of 0.001 µM. A possible explanation could be an inhibition of enzymes upstream the isoprenoid pathway. Also, carnosic acid (IC_50_ 4.2 µM) and chlorogenic acid (IC_50_ 169 µM) showed an effect on cholesterol biosynthesis, without affecting the isoprenoid pathway. For the inhibitor of HMG‐CoA reductase (**D**), simvastatin, and the inhibitors of lanosterol 14α‐demethylase (**N**), clotrimazole, and voriconazole, IC_50_ values between 0.2 and 45 µM were determined.

**Table 3:** Characterization of cholesterol biosynthesis inhibitors.

	Qualitative screening		Quantitative screening		
Inhibitor	Postulated inhibited enzyme	Inhibited enzyme	IC_50_ value [µM]	Confidence interval^a^ [µM]	R^2^
Bempedoic acid	**A**	‐	>1000	‐	‐
Simvastatin	**D**	‐	0.580	0.361–0.930	0.948
6‐Fluoromevalonate	**G, H**	**H**	>1000	‐	‐
BPH‐1358	I	**‐**	n.q.	‐	‐
Carnosic acid	**I**	‐	4.2	3.1–5.7	0.973
Zoledronic acid	**I**	I	**225**	176–289	0.951
YM‐175	**I**	**I**	337	264–432	0.965
“Carbazole 11”	**J**	‐	0.001	0.001–0.002	0.990
Lapaquistat	**J**	**J**	2.8	2.0–3.8	0.975
Zaragozic acid	**J**	J	5.0	1.3–18.4	0.983
Chlorogenic acid	**J**	‐	169	141–201	0.983
NB‐598	**L**	**L**	0.006	0.003–0.013	0.986
Terbinafine	**L**	**L**	0.540	0.305–0.958	0.964
Naftifine	**L**	**L**	1.9	1.6–2.4	0.987
Clotrimazole	**N**	**N**	0.202	0.132–0.310	0.965
Voriconazole	**N**	**N**	45	33–61	0.937

*Note*: Target enzymes: **A**, ATP citrate synthase; **D**, HMG‐CoA reductase; **G**, mevalonate pyrophosphate decarboxylase; **H**, isopentenyl pyrophosphate isomerase; **I**, farnesyl pyrophosphate synthase; **J**, squalene synthase; **L**, squalene epoxidase; **N**, lanosterol 14α‐demethylase.

Abbreviations: ‐, not determined/applicable; n.q, not quantified.

^a^ Confidence interval for the IC_50_ value was 95%; R^2^ linear fit.

#### 2.3.2 Characterization of isoprenoids

In all samples where an accumulation of isoprenoid pyrophosphates was detected, also the corresponding isoprenoid was detected (see Section 2.3.3). The highest concentration of pyrophosphates was determined in the samples of 6‐fluoromevalonate, with 88% of the measured isoprenol *tert*‐butyldiphenylsilyl (*t*BDPS) ether derived from IPP. Considering pyrophosphate distribution of IPP, the major amount of IPP was measured intracellularly, with only 27% of the IPP detected in the extracellular matrix (Table 4, for quantification results [ng/sample] see Supporting Information S1: Table S1). In samples from both NBPs, zoledronic acid and YM‐175 three different pyrophosphates were detected. Next to the two C_5_ isomers IPP and DMAPP, also the C_10_ intermediate geranyl pyrophosphate (GPP) was identified. Comparing the two NBPs, a higher concentration of pyrophosphorylated isoprenoids was determined in the samples of YM‐175 where 68% of the measured isoprenol *t*BDPS ether and 40% of prenol *t*BDPS ether originated from IPP and DMAPP, respectively. For the quantified geraniol *t*BDPS ether, 10% were attributed to GPP. Zoledronic acid samples only contained isoprenoid pyrophosphates in a range from 22% to 29%, of which 78%–100% were present in the cellular matrix. From YM‐175 samples 60%–79% of the detected isoprenoid pyrophosphates were present intracellularly. Further compounds inducing an accumulation of pyrophosphorylated isoprenoids were the two SQS inhibitors ZA and lapaquistat. In samples from both inhibitors, FPP was detected. While in lapaquistat samples 23% of the measured farnesol *t*BDPS ether originated from FPP, in the ZA samples only 2% of the farnesol *t*BDPS ether originated from FPP. Samples from lapaquistat additionally contained the geranylgeraniol *t*BDPS ether, of which 33% originated from GGPP. In lapaquistat samples, the predominant fractions of FPP and GGPP, 65% and 68%, were present in cellular matrix. From ZA samples, FPP was only identified in the cellular matrix.

**Table 4:** Analyte ratio: Heatmap giving the relative amount of isoprenoid pyrophosphates in a ratio to the measured analytes per sample (free + deconjugated alcohols) of the whole sample (cellular + extracellular matrix).

		Analyte ratio pyrophosphate/isoprenoid					Pyrophosphate distribution intra/extracellular								
Inhibitor	Concen‐tration [µM]	IPP	DMAPP	GPP	FPP	GGPP	IPP	DMAPP	GPP	FPP	GGPP				
Bempedoic acid	500	n.q.	n.q.	n.q.	n.q.	n.q.	n.q.	n.q.	n.q.	n.q.	n.q.	isoprenoid 100%			intracellular 100%
Simvastatin	1	n.q.	n.q.	n.q.	n.q.	n.q.	n.q.	n.q.	n.q.	n.q.	n.q.				
6‐Fluoromevalonate	500		n.q.	n.q.	n.q.	n.q.		n.q.	n.q.	n.q.	n.q.				
BPH‐1358	50	n.q.	n.q.	n.q.	n.q.	n.q.	n.q.	n.q.	n.q.	n.q.	n.q.				
Carnosic acid	50	n.q.	n.q.	n.q.	n.q.	n.q.	n.q.	n.q.	n.q.	n.q.	n.q.				
YM‐175	100				n.q.	n.q.				n.q.	n.q.				
Zoledronic acid	500				n.q.	n.q.				n.q.	n.q.				
“Carbazole 11”	1	n.q.	n.q.	n.q.	n.q.	n.q.	n.q.	n.q.	n.q.	n.q.	n.q.				
Chlorogenic acid	500	n.q.	n.q.	n.q.	n.q.	n.q.	n.q.	n.q.	n.q.	n.q.	n.q.				
Lapaquistat	50	n.q.	n.q.	n.q.			n.q.	n.q.	n.q.			100% pyrophosphate			100% extracellular
Zaragozic acid	10	n.q.	n.q.	n.q.		n.q.	n.q.	n.q.	n.q.		n.q.				
Naftifine	1	n.q.	n.q.	n.q.	n.q.	n.q.	n.q.	n.q.	n.q.	n.q.	n.q.				
NB‐598	50	n.q.	n.q.	n.q.	n.q.	n.q.	n.q.	n.q.	n.q.	n.q.	n.q.				
Terbinafine	1	n.q.	n.q.	n.q.	n.q.	n.q.	n.q.	n.q.	n.q.	n.q.	n.q.				
Clotrimazole	1	n.q.	n.q.	n.q.	n.q.	n.q.	n.q.	n.q.	n.q.	n.q.	n.q.				
Voriconazole	500	n.q.	n.q.	n.q.	n.q.	n.q.	n.q.	n.q.	n.q.	n.q.	n.q.				

*Note*: Red color implements a high amount of isoprenoid pyrophosphates, while green color implements a high amount of the free isoprenoid. Blue color indicates an equal distribution between pyrophosphates and alcohols in the sample. Pyrophosphate distribution: Heatmap displaying the distribution of pyrophosphates between the matrices. Purple color indicates a high concentration of pyrophosphates in the extracellular matrix while orange color indicates a preferred intracellular distribution. The tested concentration is a maximum nontoxic concentration.

Abbreviation: n.q., not quantified (<LLOQ).

#### 2.3.3 Isoprenoid trafficking: Analyte distribution between the intra‐ and extracellular matrix

The analyte distribution of the isoprenoids (deconjugated + free isoprenoids) and squalene between the intra‐ and extracellular matrix are visualized in Table 5 (analyte distribution intra‐/extracellular). In addition, the absolute amounts of analytes per sample (sum of extracellular + cellular matrix) are shown in Table 5. Results [ng/sample] can be found in Supporting Information S1: Table S1. Basal levels of all isoprenoids were below the LLOQ (2.5 ng/mL). Only squalene was quantified in untreated matrix samples with concentrations of approx. 76 ng/sample in cellular matrix samples and approx. A total of 15 ng/sample in extracellular matrix samples.

**Table 5:** Analyte distribution: Heatmap depicting the ratio between intracellular and extracellular concentration of the detected isoprenoids.

			Analyte distribution intra/extracellular						Absolute amount of analyte										
Inhibitor	Concen‐tration [µM]	Postulated target enzyme	Isoprenol	Prenol	Geraniol	Squalene	Farnesol	Geranylgeraniol	Isoprenol	Prenol	Geraniol	Squalene	Farnesol	Geranylgeraniol					
Bempedoic acid	500	**A**	n.q.	n.q.	n.q.			n.q.	n.q.	n.q.	n.q.			n.q.	intracellular 100%			1000	ng/sample
Simvastatin	1	**D**	n.q.	n.q.	n.q.		n.q.	n.q.	n.q.	n.q.	n.q.		n.q.	n.q.					
6‐Fluoromevalonate	500	**G, H**		n.q.	n.q.		n.q.	n.q.		n.q.	n.q.		n.q.	n.q.					
BPH‐1358	50	**I**		n.q.	n.q.		n.q.	n.q.		n.q.	n.q.		n.q.	n.q.					
Carnosic acid	50		n.q.	n.q.	n.q.		n.q.	n.q.	n.q.	n.q.	n.q.		n.q.	n.q.					
YM‐175	100						n.q.	n.q.					n.q.	n.q.				100	
Zoledronic acid	500						n.q.	n.q.					n.q.	n.q.					
“Carbazole 11”	1	**J**			n.q.		n.q.	n.q.			n.q.		n.q.	n.q.					
Chlorogenic acid	500		n.q.	n.q.	n.q.		n.q.	n.q.	n.q.	n.q.	n.q.		n.q.	n.q.					
Lapaquistat	50		n.q.	n.q.	n.q.				n.q.	n.q.	n.q.								
Zaragozic acid	10		n.q.	n.q.	n.q.				n.q.	n.q.	n.q.				100% extracellular			10	
Naftifine	1	**L**	n.q.	n.q.	n.q.		n.q.	n.q.	n.q.	n.q.	n.q.		n.q.	n.q.					
NB‐598	50			n.q.	n.q.			n.q.		n.q.	n.q.			n.q.					
Terbinafine	1		n.q.	n.q.	n.q.		n.q.	n.q.	n.q.	n.q.	n.q.		n.q.	n.q.					
Clotrimazole	1	**N**	n.q.	n.q.	n.q.		n.q.	n.q.	n.q.	n.q.	n.q.		n.q.	n.q.					
Voriconazole	500		n.q.	n.q.	n.q.		n.q.	n.q.	n.q.	n.q.	n.q.		n.q.	n.q.				1	

*Note*: Orange color indicates a high ratio of intracellular intermediates, while purple color indicates a high ratio of extracellular intermediates. Blue color indicates an equal distribution in both matrices. Absolute amount of analyte: Heatmap depicting the absolute amount of isoprenoid *t*BDPS ethers and squalene quantified in the whole samples (cellular + extracellular matrix). **A**, ATP citrate synthase; **D**, HMG‐CoA reductase; **G**, mevalonate pyrophosphate decarboxylase; **H**, isopentenyl pyrophosphate isomerase; **I**, farnesyl pyrophosphate synthase; **J**, squalene synthase; **L**, squalene epoxidase; **N**, lanosterol 14α‐demethylase. Values are in ng/sample. The tested concentration is the maximum nontoxic concentration.

Abbreviations: n.q., not quantified (<LLOQ); tBDPS, *tert*‐butyldiphenylsilyl.

Isoprenol was detected in samples from six different inhibitors. The highest concentration of isoprenol was determined in samples from 6‐fluoromevalonate, the direct inhibitor of IPP isomerase (**H**), followed by both FPPS (**I**) inhibitors, YM‐175 and zoledronic acid (33–310 ng/sample). Furthermore, traces of isoprenol (10 ng/sample) were identified in samples from BPH‐1358 (postulated inhibitor of **I**), “carbazole 11” (postulated inhibitor of **J**) and NB‐598 (postulated inhibitor of **L**). While high concentrations were distributed equally between the two matrices showing only a slight tendency toward the intracellular matrix, low concentrations were only measured intracellular (BPH‐1358, “carbazole 11”) or extracellular (NB‐598). Only three inhibitors (YM‐175, zoledronic acid, and “carbazole 11”) induced an accumulation of prenol. The maximum amount of prenol (480 ng/sample) was observed in samples from YM‐175, followed by zoledronic acid (90 ng/sample) and “carbazole 11” (22 ng/sample). While in samples of the two NBPs (YM‐175 and zoledronic acid) the extracellular prenol content was higher, in “carbazole 11,” prenol was equally distributed. Additionally, geraniol was also detected in zoledronic acid (27 ng/sample) and in YM‐175 (124 ng/sample) samples. It is noteworthy that a significant proportion of geraniol was identified in extracellular matrix, with only minimal amounts detected in the cellular matrix. Squalene was quantified in all samples. The approximate concentration of squalene was between 30 and 50 ng in all samples, that were not affected by SQS (**J**) or squalene epoxidase (SE, **L**) inhibitors. Only samples from bempedoic acid (inhibitor of **A**) contained a lower amount of squalene (27 ng/sample) while samples from BPH‐1358 contained a slightly higher amount (55 ng/sample). Both SQS inhibitors ZA and lapaquistat induced a reduction of squalene to approx. 25 ng/sample, whereas “carbazole 11” and chlorogenic acid did not decrease the squalene content. Samples from chlorogenic acid even contained an increased squalene content (56 ng/sample). All three SE inhibitors increased the squalene concentration >60 ng/sample. NB‐598 was the most effective one, with more than 600 ng of squalene quantified in the respective samples. The effects of terbinafine and naftifine were almost 10‐fold lower with amounts of 62 and 73 ng/sample, respectively. Squalene distribution between matrices depended on the used inhibitor. The effective SQS inhibitors ZA and lapaquistat decrease the intracellular squalene level so that a major part of the detected squalene was measured extracellularly. A very similar isoprenoid pattern was determined from bempedoic acid samples, where a major part of squalene was measured from extracellular matrix. The distribution of squalene due to SE inhibitors resulted in an intracellular accumulation of squalene. Similar observations were determined for the two NBPs (YM‐175 and zoledronic acid) and simvastatin. Four inhibitors led to an accumulation of farnesol in quantifiable concentrations. Approximately 2000 ng farnesol per sample were detected in samples treated with ZA or lapaquistat. In addition, traces of farnesol were measured in samples treated with bempedoic acid and NB‐598, with concentrations below 10 ng/sample. While farnesol was equally distributed between both matrices in samples from lapaquistat, the majority of farnesol was measured in the extracellular matrix of the remaining samples. Geranylgeraniol was only quantified in samples from the two SQS inhibitors, ZA (24 ng/sample) and lapaquistat (52 ng/sample). In contrast to the other isoprenoids, a substantial portion of geranylgeraniol was found in extracellular matrix.

## 3 CONCLUSION

In this work, we present a fully validated gas chromatography‐mass spectrometry (GC‐MS) approach for the analysis of isoprenoids and isoprenoid pyrophosphates from cellular samples. Next to the cellular matrix, the corresponding extracellular matrix was analyzed. The high sensitivity (2.5 ng/mL for all isoprenoids, see Tables 1 and 2) of our method allows us to identify even minor alterations in the isoprenoid pattern. Furthermore, the broad calibration range (upper limit of 1000 ng/mL for all analytes, see Tables 1 and 2) allows us to quantify the extent of inhibitor‐induced accumulations. Another advantage of the method is the high precision not exceeding a standard deviation of 15% over all samples and concentrations (see Tables 1 and 2). The recovery of the isoprenoid pyrophosphates underlines our findings from prior analyses^[^ ^36^ ^]^ and gives constant results within the measurements with SD values below 8% at all concentrations above the LLOQ.

As indicated by their IC_50_ values, the majority of inhibitors, with the exception of bempedoic acid, 6‐fluoromevalonate (both IC_50_ >1000 µM), and BPH‐1358 (no IC_50_ determined), had an impact on total cholesterol biosynthesis (Table 3). The lack of activity observed with bempedoic acid, an approved drug for the treatment of hyperlipidemia, can be attributed to the absence of the prodrug activation in our HL60 cell model. Given that the requisite enzyme, very long‐chain acyl‐CoA synthetase (EC 6.2.1.3), exhibits high tissue specificity, the drug is only activated in liver cells.^[^ ^12^ ^]^

The high separation efficiency of GC‐MS systems enabled us to distinguish between the two C_5_ isomers prenol and isoprenol, which is normally not possible in liquid chromatography‐mass spectrometry (LC‐MS) approaches. Therefore, we revealed an effect of 6‐fluoromevalonate on the enzyme IPP isomerase (**H**), which, to the best of our knowledge, has not been previously described in the literature. However, 6‐fluoromevalonate seems to act as a multienzyme inhibitor, due to its additional effect on the upstream enzyme mevalonate pyrophosphate decarboxylase (**G**).^[^ ^16^ ^]^ Henneman et al.^[^ ^17^ ^]^ explained the accumulation of further mevalonate pathway intermediates by feedback mechanisms and reversible reactions. However, they could not explain whether the accumulation of C_5_ isoprenoids was due to direct inhibition of IPP isomerase (**H**)/FPPS (**I**) or a feedback mechanism. Our findings support the hypothesis of a direct inhibition of IPP isomerase (**I**) by the selective accumulation of isoprenol and IPP in our experiments involving 6‐fluoromevalonate.

Further, we could not confirm the effects of some postulated pre‐squalene pathway enzyme inhibitors, namely carnosic acid, which was expected to target FPPS (**I**), as well as “carbazole 11” and chlorogenic acid that were described as SQS (**K**) inhibitors even though an inhibitory effect on total sterol biosynthesis was observed for all of them (Table 3). However, neither the isoprenoid pathway, nor the post‐squalene pathway were affected by them. Therefore, an inhibition of enzymes upstream the isoprenoid pathway must be considered.

The analysis of the pyrophosphate patterns (Table 4) revealed that most samples contained free isoprenoids next to pyrophosphorylated isoprenoids in similar concentrations after enzymatic inhibition. This was surprising because the pyrophosphorylated isoprenoids are the direct biosynthesis intermediates and were therefore expected to accumulate. Only samples treated with 6‐fluoromevalonate and YM‐175 (Table 4) contained IPP, the pyrophosphorylated form of isoprenol, in a higher amount than the free alcohol. The matrix distribution pattern of the pyrophosphorylated isoprenoids further revealed that all pyrophosphorylated analytes have a strong tendency to distribute into cellular matrix. Only low quantities of pyrophosphorylated analytes were identified in the corresponding extracellular matrix samples, which could be explained by a lower permeability of isoprenoid pyrophosphates through the cellular membrane.

The additional analysis of the total isoprenoid pattern, including the free alcohols next to the pyrophosphorylated isoprenoids (Table 5), allowed us to analyze the isoprenoid trafficking in more detail. Overall, free isoprenoids seemed to be equally distributed between cellular and extracellular matrix, showing slight tendencies toward the intracellular matrix. A strong preference toward the intracellular matrix was observed for the free isoprenoid geranylgeraniol. The opposite trend, toward the extracellular matrix, was observed for geraniol and farnesol (Table 5). The matrix distribution pattern of the free isoprenoids, geraniol and farnesol, is contrary to their pyrophosphate distribution pattern (Table 4). Therefore, we concluded that the deconjugated forms of geraniol and farnesol were the preferred ones to be excreted from cells in our HL60 cell model. The presence of farnesol in the extracellular matrix is, however, in contrast to previous findings from *A. fumigatus* cells,^[^ ^36^ ^]^ in which we identified FPP as the intermediate to be excreted when it comes to a downregulation of the essential *erg9* gene, which encodes for the fungal SQS enzyme (analog enzyme **K**). For this reason, analyzing the distribution patterns of isoprenoids and their corresponding pyrophosphates can help to understand potential effects, side effects, and mechanisms of action of substances targeting the isoprenoid pathway of sterol biosynthesis. In addition, the matrix distribution patterns as well as the pyrophosphate patterns can be used to point out differences between organisms using the identical isoprenoid biosynthesis pathway. The applicability of our assay toward fungal and mammalian cell matrices makes it a novel tool that can help to develop selective, organism‐specific inhibitors of sterol biosynthesis. But also, beyond sterol biosynthesis, the investigation of pyrophosphate patterns is of high interest. Due to the significant role of farnesyl and GGPP in prenylation reactions, the interest in determining their concentration, and influencing their composition is of major importance.

## 4 EXPERIMENTAL

### 4.1 Chemicals and reagents

All solvents were purchased in *pro analysis* quality or high‐performance liquid chromatography grade from Merck. Deionized water was prepared with an in‐house ion‐exchanger.

#### 4.1.1 Standards

Prenol (97%) and farnesol (97%) were purchased from Alfa Aesar. Isoprenol (98%) was from Tokyo Chemical Industry Co. Geraniol (98%), squalene (98%) as well as 5α‐cholestane (97%) (Internal Standard for sterol analysis (IS_Sterol_) were from Merck, whereas geranylgeraniol (85%) and 1‐heptadecanol (98%) (Internal Standard for isoprenoid analysis (IS_Isoprenoid_) were obtained from Merck. All three pyrophosphates (DMAPP (95%), FPP (95%), GGPP (95%)) were purchased from Cayman Chemicals. 2‐^13^C‐Acetate (99%) was purchased from Merck.

#### 4.1.2 Inhibitors

6‐Fluoromevalonate (90%), clotrimazole (98%), naftifine hydrochloride (99%), simvastatine (97%), terbinafine hydrochloride (98%), and voriconazole (98%) were purchased from Merck. Bempedoic acid (95%), BPH‐1358 (95%), carnosic acid (95%), chlorogenic acid (95%), ZA A (95%), and zoledronic acid monohydrate (95%) were obtained from Cayman Chemicals. Lapaquistat (Tak‐475; 99%) was purchased from BLD Pharmatech GmbH and NB‐598 (99%) was purchased from MCE‐MedChemExpress. “Carbazole 11” and YM‐175 were synthesized in‐house according to literature.^[^ ^25^, ^39^ ^]^

#### 4.1.3 Sample preparation

Isoprenoid assay: An alkaline buffer pH 8.6 was used for enzymatic deconjugation containing diethanolamine (DEA; 99%) from Tokyo Chemical Industry Co. and magnesium chloride hexahydrate (99%) from Merck. Bovine alkaline phosphatase (P7640) was obtained from Merck. Sodium chloride was purchased from Bernd Kraft GmbH. For derivatization of isoprenoids, imidazole (99%) and *tert*‐butyldiphenylchlorosilane (*t*BDPSCl) (98%) from Merck were used. Cholesterol assay: Phosphate‐buffered saline (PBS) tablets from Merck were used to prepare PBS buffer pH 7.4 ± 0.2. Silylation of sterols was conducted with a mixture of *N*‐methyl‐*N*‐trimethylsilyltrifluoroacetamide (MSTFA) and *N*‐trimethylsilylimidazole (TSIM; 10/1; *v*/*v*) from Macherey Nagel.

#### 4.1.4 Cell culture

HL60 cells were obtained from the German Collection of Microorganisms and Cell Cultures GmbH (DSMZ) and cultivated at 37°C in a humidified atmosphere containing 5% CO_2_ in RPMI 1640 medium with 10% fetal bovine serum (FBS) both from PAA Laboratories. Lipid‐free HL60 medium was from PAN Biotech.

### 4.2 Analytical instrument

All samples were analyzed with an Agilent 7820 A gas chromatograph coupled to a quadrupole 5977B from Agilent. For sampling a 7693 A automatic liquid sampler (ALS) combined with the G4513A split/splitless injector from Agilent was used. Chromatography was performed on an HP‐5ms Ultra Inert (30 m × 0.25 mm × 0.25 µm) capillary column. The carrier gas was helium 5.0 from AIR Liquide used at a constant flow rate of 1.2 mL/min. For the analysis of isoprenoid *t*BDPS ethers, inlet temperature was kept at 270°C throughout the whole GC run and the injection volume was 1 µL. Initial oven temperature was set to 75°C, which was held for 0.5 min before it ramped to 180°C with a heat rate of 25°C/min. After a hold time of 1.0 min temperature was increased to 225°C with a heating rate of 15°C/min followed by a third ramp of 50°C/min up to 320°C, where the column was held for 5.9 min. For postrun, the flow rate was increased to 2.0 mL/min for 2.5 min, which makes a total run time of 19.0 min. Transfer line temperature was permanently set at 270°C. The 5977B single quadrupole was operated in single ion monitoring (SIM) mode at 70 eV after a solvent delay of 9.5 min (see details in Table 6). The ion source temperature was set at 230°C and the quadrupole temperature at 150°C. Instrument control and data analysis were performed with Agilent Masshunter 8.0 software from Agilent.

**Table 6:** Analytical details of the analyzed isoprenoid *t*BDPS ethers and squalene.

Trivial name	RT [min]	RRT (IS_Isoprenoid_)	Qualifier and quantifier ions [*m/z*]
Isoprenol	9.781	0.710	189, **225**, 267
Prenol	9.845	0.715	69, 189, **267**
Geraniol	11.243	0.816	69, **335**, 392
Squalene	11.918	0.865	**69**, 81, 410
Farnesol	12.578	0.913	**69**, 203, 403
1‐Heptadecanol (IS_Isoprenoid_)	13.775	1.000	71, 123, **437**
Geranylgeraniol	14.968	1.087	**69**, 81, 471

*Note*: Absolute retention time (RT); relative retention time (RRT); in bold quantifier ions.

Abbreviation: *t*BDPS, *tert*‐butyldiphenylsilyl.

### 4.3 Sample preparation

#### 4.3.1 General sample preparation of cellular and extracellular matrix

The incubation of HL60 cells with test substances was conducted in 24‐well plates. In each well 1 × 10^6^ cells per milliliters were seeded according to the protocol by Müller et al.^[^ ^31^ ^]^ The inhibitor solutions were prepared by dissolving the respective substances in ethanol, dimethyl sulfoxide, or 0.1 M aqueous sodium hydroxide solution. Stock solutions of each substance were prepared and diluted in a lipid‐free HL60 medium to the final test concentrations. For quantitative screening, an additional 2‐^13^C‐acetate solution (6.25 mg/mL) in purified water was added, followed by a 24 ± 2 h incubation period in a 37°C humidified atmosphere containing 5% CO_2_.

#### 4.3.2 Sample preparation procedure for the identification of target enzymes within the pre‐squalene pathway

For initial qualitative screening of potential inhibitors, two distinct test concentrations were used (1 and 50 µM). For the analysis of HL60 cells, the suspended cells from each well were transferred into a 2.0 mL microcentrifuge safe‐lock tube and centrifuged for 5 min at 600 *g* at room temperature (RT). After centrifugation, the supernatant (extracellular matrix) was decanted from the remaining cell pellet (cellular matrix). For further analysis of the extracellular matrix, the aqueous solution was lyophilized before being processed equivalent to cellular samples. The cell pellet/lyophilized medium was resuspended in 590 µL DEA‐buffer before it was mechanically lysed (only cellular samples) for 5 min on a vortex shaker using three 1.5 mm and three 3.0 mm glass beads. Subsequently, 10 µL of bovine alkaline phosphatase suspension in DEA‐buffer (40 mg/mL) were added and the samples were shaken vigorously for 1 min by hand before they were incubated for 40 min at 37°C. Enzymatic deconjugation was terminated by the addition of 300 ± 6 mg NaCl, 300 µL of acetonitrile/acetone (2/1; *v*/*v*), 350 µL of *n*‐hexane, and 100 µL of an internal standard mixture containing 1‐heptadecanol (50 µg/mL, IS_Isoprenol_) and cholestane (10 µg/mL, IS_Sterol_) in *n*‐hexane. The samples were shaken by hand for 1 min before they were centrifuged for 5 min at 12,000 *g* at RT. After centrifugation, 450 µL of the upper, *n*‐hexane layer were transferred into a GC‐vial (first extraction step). For a second extraction, 750 µL of *n*‐hexane were added before samples were shaken by hand for 1 min before they were centrifuged for 5 min at 12,000 *g* at RT. After centrifugation, 650 µL of the organic upper layer were combined with the extract from the first extraction. For complete derivatization, the combined organic extracts (1100 µL) were stored for 30 min at 70°C after 30 µL of *tert*‐butyldiphenylchlorosilane (*t*BDPSCl) and 30 µL of imidazole solution (262 mg/mL in tetrahydrofuran) were added. Derivatization was essential to increase the retention times of the volatile isoprenoids and to distinguish between the two C_5_ isomers. The derivatized samples were subjected to GC‐MS analysis.

#### 4.3.3 Determination of IC_50_ values for inhibition of total cholesterol biosynthesis

IC_50_ values were determined on total cholesterol biosynthesis, to generate comparable results unaffected by a possible multienzyme inhibition. To quantify *de novo* synthesized cholesterol, the protocol of Müller et al.^[^ ^31^ ^]^ was used.

### 4.4 Method validation

Method validation was performed in accordance with the EMA guideline on bioanalytical method validation EMEA/CHMP/EWP/192217/2009,^[^ ^37^ ^]^ considering the parameters linearity and LLOQ, matrix effects, accuracy, precision, and recovery. Method validation was performed on HL60 cells and on the HL60 medium. The HL60 medium was lyophilized before use. In all validation tests (besides recovery), the free isoprenoids were analyzed.

#### 4.4.1 Linearity and LLOQ

For determination of linearity, sample matrix (cells or lyophilized medium) was spiked with analyte stock solutions of 11 different concentrations giving final concentrations of 1.0, 2.5, 5.0, 7.5, 10, 25, 50, 100, 250, 500, and 1000 ng/mL. All samples were prepared in triplicates and contained internal standard (IS_Isoprenoid_) in a consistent concentration (5 µg/mL). In addition, nonspiked matrix blanks, only containing IS_Isoprenoid_ were analyzed. Signal area ratios from the analyte and IS_Isoprenoid_ quantifier ions were plotted against the corresponding analyte concentration. The calibration curve was weighted 1/*x*. The LLOQ was defined as the lowest concentration of the calibration curve which could be quantified reliably.

#### 4.4.2 Matrix effects

MF were investigated at low (3 × LLOQ) and high (1000 ng/mL) concentrations, by comparing spiked samples to samples of the same concentrations in *n*‐hexane (*n *= 6). The EMA guideline recommends the use of blank matrix, which was not conductible in this case, due to the endogenous content of squalene in cellular samples, which could indicate false positive matrix effects for squalene. Therefore, the pooled matrix was used in both (cells and medium) approaches. As an indicator for matrix effects, the matrix factor was calculated as the quotient of the signal area in the matrix to signal area in *n*‐hexane. In addition, the relative standard deviation (RSD) at both concentrations was calculated which should be ≤15% according to the guideline.

#### 4.4.3 Method accuracy and precision

Method accuracy and method precision were determined at four different levels: the individual LLOQs, low (≤3 × LLOQ), medium (500 ng/mL), and high (1000 ng/mL) concentrations. Quality control (QC) samples of different concentrations were prepared by spiking previously prepared pooled sample matrix with an appropriate stock solution containing a mixture of all analytes before derivatization (*n* = 6). Signal areas of the QC samples were used to back‐calculate their concentration using a calibration curve generated from calibration standards that were prepared independent of the QC standards. Finally, the back‐calculated concentration was divided by the nominal concentration. Precision was expressed as the RSD of the QC samples at each of their four concentration levels (LLOQ, low, medium, high; *n* = 6).

#### 4.4.4 Recovery of isoprenoids from isoprenoid pyrophosphates

To determine the recovery of isoprenoids originating from their respective isoprenoid pyrophosphates, three representative pyrophosphates were used: DMAPP (C_5_), FPP (C_15_), and GGPP (C_20_). The pyrophosphates were used in equimolar concentrations to the LLOQ, low, medium, and high concentrations of the free isoprenoids. All samples were spiked with adequate pyrophosphate solutions previous to the resuspension step. Recovery was calculated as the quotient of normalized pyrophosphate sample areas and normalized matrix‐matched QC standard areas (*n* = 6).

### 4.5 Biological tests

#### 4.5.1 Characterization of inhibitors

In a qualitative screening, all inhibitors were initially tested at two concentrations (1 and 50 µM) to determine their capability to inhibit enzymes of the isoprenoid pathway. Next to the qualitative screening, every active compound, which showed an inhibition in the cholesterol biosynthesis, was subjected to a quantitative analysis. Therefore, an IC_50_ value on total cholesterol biosynthesis was determined, by incubating HL60 cells in the presence of 2‐^13^C‐acetate. Thereby, newly synthesized cholesterol can be distinguished from existing cholesterol within the matrix. Every inhibitor was tested in triplicates at several concentrations to determine a sigmoidal dose‐response curve. Furthermore, a Bradford assay^[^ ^40^ ^]^ was conducted to quantify protein content which was used for normalization and identification of potentially toxic concentrations (for details see Müller et al.^[^ ^31^ ^]^).

#### 4.5.2 Isoprenoid trafficking—ratio between isoprenoid pyrophosphates and corresponding isoprenoids

To distinguish between isoprenoid pyrophosphates and their corresponding isoprenoids, the sample preparation was modified. Two samples from each of the six biological replicates were combined. The combined samples were then split equally to create two batches of samples with an equal composition (each batch *n *= 3) that could be prepared with/without the enzymatic deconjugation step (see Section 4.3.2). After following the remaining sample preparation procedure (see Section 4.3.2), this adaption allows us to distinguish intermediates originating from pyrophosphates from the corresponding free isoprenoids. When the enzymatic step was conducted, the free alcohols and the deconjugated alcohols were measured as a sum. Whereas if the enzymatic step was not included, only the free alcohols were measured. The respective extracellular matrix of every sample was treated equivalent to the cellular sample.

According to this method, the free isoprenoid and isoprenoid pyrophosphate amounts were determined in the extracellular and cellular matrix. The whole sample analyte amount was defined as the sum of isoprenoid and isoprenoid pyrophosphate from one sample (sum of extracellular and cellular matrix). Therefore, the portion of isoprenoid pyrophosphate per sample was the quotient of isoprenoid pyrophosphate and the whole sample analyte amount of one sample. A value close to 1 was equivalent to a high isoprenoid pyrophosphate (low isoprenoid) content, whereas a value close to 0 was equivalent to a low pyrophosphate (high isoprenoid) content. The pyrophosphate distribution was the quotient of cellular isoprenoid pyrophosphate and whole sample isoprenoid pyrophosphate (sum of extracellular and cellular matrix). A value close to 1 was equivalent to a high cellular (low extracellular) content, whereas a value close to 0 was equivalent to a low cellular (high extracellular) isoprenoid pyrophosphate distribution.

Inhibitor concentrations were considered as the maximum concentration which did not influence protein content (determined by Bradford assay). All isoprenoids were measured as their corresponding *t*BDPS ethers.

#### 4.5.3 Isoprenoid trafficking—distribution of the analytes between intracellular and extracellular compartments

To investigate the distribution of the analytes between the cellular and extracellular matrix, the total analyte amount (sum of isoprenoid pyrophosphate and isoprenoid) of the cellular samples was compared with the content of the whole sample amount (extracellular + cellular matrix). A value close to 1 was equivalent to a high cellular (low extracellular) content, whereas a value close to 0 was equivalent to a low cellular (high extracellular) analyte distribution. In addition, the whole sample amount was displayed.

## CONFLICTS OF INTEREST STATEMENT

The authors declare no conflicts of interest.

## Supporting information

Supporting information.

## Data Availability

The data that support the findings of this study are available on request from the corresponding author. The data are not publicly available due to privacy or ethical restrictions.
