# Supplementary material for: Targeting the isoprenoid pathway in choleste biosynthesis: An approach to identify isoprenoid biosynthesis inhibitors
Source: Arch Pharm (Weinheim). 2025 Feb 13;358(2):e2400807. doi: 10.1002/ardp.202400807 (PMC11823600; doi:10.1002/ardp.202400807)
Supplement: Supplementary file 1 — Supporting information. [file ARDP-358-e2400807-s001.docx]

Targeting the isoprenoid pathway in cholesterol biosynthesis – An approach to identify isoprenoid biosynthesis inhibitors

Maximilian Liebl^1^, Florian Olander^1^ and Christoph Müller^1^*

1 Department of Pharmacy - Center for Drug Research, Ludwig-Maximilians-Universität München, Munich, Germany

*Correspondence:

Christoph Müller, Department of Pharmacy - Center for Drug Research, Ludwig-Maximilians-Universität München, Butenandtstraße 5-13, 81377 München, Germany

Email: [christoph.mueller@cup.uni-muenchen.de](mailto:christoph.mueller@cup.uni-muenchen.de)

**Table S1.** Absolute concentration of analyte tBDPS ethers and squalene per sample. + enzyme covers free isoprenoids and deconjugated isoprenoids. – enzyme covers free isoprenoids. The tested concentration is a maximum non-toxic concentration. n.q. not quantified (<LLOQ).

| inhibitor | concen-tration [µM] | **ng/sample** | | | | | | | | | | | |  |
| --- | --- | --- | --- | --- | --- | --- | --- | --- | --- | --- | --- | --- | --- | --- |
|  |  | **cellular matrix** | | | | | | | | | | | |  |
|  |  | + enzyme | | | | | | - enzyme | | | | | |  |
|  |  | isoprenol | prenol | geraniol | squalene | farnesol | geranylgeraniol | isoprenol | prenol | geraniol | squalene | farnesol | geranylgeraniol |  |
|  |  |  |  |  |  |  |  |  |  |  |  |  |  |  |
|  |  |  |  |  |  |  |  |  |  |  |  |  |  |  |
|  |  |  |  |  |  |  |  |  |  |  |  |  |  |  |
|  |  |  |  |  |  |  |  |  |  |  |  |  |  |  |
| bempedoic acid | 500 | n.q. | n.q. | n.q. | 10 | n.q. | n.q. | n.q. | n.q. | n.q. | 14 | n.q. | n.q. |  |
| simvastatin | 1 | n.q. | n.q. | n.q. | 31 | n.q. | n.q. | n.q. | n.q. | n.q. | 24 | n.q. | n.q. |  |
| 6-fluoromevalonate | 500 | 208 | n.q. | n.q. | 18 | n.q. | n.q. | 21 | n.q. | n.q. | 16 | n.q. | n.q. |  |
| BPH-1358 | 50 | 3 | n.q. | n.q. | 33 | n.q. | n.q. | 3 | n.q. | n.q. | 29 | n.q. | n.q. |  |
| carnosic acid | 50 | n.q. | n.q. | n.q. | 23 | n.q. | n.q. | n.q. | n.q. | n.q. | 23 | n.q. | n.q. |  |
| YM-175 | 100 | 103 | 199 | 17 | 28 | n.q. | n.q. | 18 | 47 | 9 | 25 | n.q. | n.q. |  |
| zoledronic acid | 500 | 18 | 38 | 6 | 36 | n.q. | n.q. | 9 | 20 | n.q. | 52 | n.q. | n.q. |  |
| „carbazole 11“ | 1 | 6 | 12 | n.q. | 36 | n.q. | n.q. | 5 | 12 | n.q. | 35 | n.q. | n.q. |  |
| chlorogenic acid | 500 | n.q. | n.q. | n.q. | 38 | n.q. | n.q. | n.q. | n.q. | n.q. | 27 | n.q. | n.q. |  |
| lapaquistat | 50 | n.q. | n.q. | n.q. | 9 | 896 | 40 | n.q. | n.q. | n.q. | 15 | 607 | 29 |  |
| zaragozic acid | 10 | n.q. | n.q. | n.q. | 13 | 516 | 16 | n.q. | n.q. | n.q. | 13 | 471 | 17 |  |
| naftifine | 1 | n.q. | n.q. | n.q. | 56 | n.q. | n.q. | n.q. | n.q. | n.q. | 79 | n.q. | n.q. |  |
| NB-598 | 50 | n.q. | n.q. | n.q. | 585 | n.q. | n.q. | n.q. | n.q. | n.q. | 549 | n.q. | n.q. |  |
| terbinafine | 1 | n.q. | n.q. | n.q. | 47 | n.q. | n.q. | n.q. | n.q. | n.q. | 53 | n.q. | n.q. |  |
| clotrimazole | 1 | n.q. | n.q. | n.q. | 26 | n.q. | n.q. | n.q. | n.q. | n.q. | 24 | n.q. | n.q. |  |
| voriconazole | 500 | n.q. | n.q. | n.q. | 13 | n.q. | n.q. | n.q. | n.q. | n.q. | 19 | n.q. | n.q. |  |
|  |  | **extracellular matrix** | | | | | | | | | | | |  |
| bempedoic acid | 500 | n.q. | n.q. | n.q. | 16 | 7 | n.q. | n.q. | n.q. | n.q. | 23 | 7 | n.q. |  |
| simvastatin | 1 | n.q. | n.q. | n.q. | 10 | n.q. | n.q. | n.q. | n.q. | n.q. | 7 | n.q. | n.q. |  |
| 6-fluoromevalonate | 500 | 102 | n.q. | n.q. | 15 | n.q. | n.q. | 33 | n.q. | n.q. | 10 | n.q. | n.q. |  |
| BPH-1358 | 50 | n.q. | n.q. | n.q. | 21 | n.q. | n.q. | n.q. | n.q. | n.q. | 11 | n.q. | n.q. |  |
| carnosic acid | 50 | n.q. | n.q. | n.q. | 16 | n.q. | n.q. | n.q. | n.q. | n.q. | 20 | n.q. | n.q. |  |
| YM-175 | 100 | 105 | 281 | 107 | 11 | n.q. | n.q. | 48 | 240 | 103 | 14 | n.q. | n.q. |  |
| zoledronic acid | 500 | 15 | 52 | 21 | 10 | n.q. | n.q. | 15 | 50 | 20 | 18 | n.q. | n.q. |  |
| „carbazole 11“ | 1 | n.q. | 10 | n.q. | 14 | n.q. | n.q. | 4 | 10 | n.q. | 7 | n.q. | n.q. |  |
| chlorogenic acid | 500 | n.q. | n.q. | n.q. | 19 | n.q. | n.q. | n.q. | n.q. | n.q. | 25 | n.q. | n.q. |  |
| lapaquistat | 50 | n.q. | n.q. | n.q. | 15 | 1026 | 12 | n.q. | n.q. | n.q. | 63 | 871 | 6 |  |
| zaragozic acid | 10 | n.q. | n.q. | n.q. | 13 | 1497 | 8 | n.q. | n.q. | n.q. | 18 | 1498 | 7 |  |
| naftifine | 1 | n.q. | n.q. | n.q. | 17 | n.q. | n.q. | n.q. | n.q. | n.q. | 25 | n.q. | n.q. |  |
| NB-598 | 50 | 6 | n.q. | n.q. | 20 | 6 | n.q. | 10 | n.q. | n.q. | 21 | 4 | n.q. |  |
| terbinafine | 1 | n.q. | n.q. | n.q. | 15 | n.q. | n.q. | n.q. | n.q. | n.q. | 22 | n.q. | n.q. |  |
| clotrimazole | 1 | n.q. | n.q. | n.q. | 14 | n.q. | n.q. | n.q. | n.q. | n.q. | 21 | n.q. | n.q. |  |
| voriconazole | 500 | n.q. | n.q. | n.q. | 20 | n.q. | n.q. | n.q. | n.q. | n.q. | 17 | n.q. | n.q. |  |
